# Mouse Germ Cell Development *in-vivo* and *in-vitro*

**Published:** 2007-06-06

**Authors:** Deshira Saiti, Orly Lacham-Kaplan

**Affiliations:** Monash Immunology and Stem Cell Laboratories, Level 3, STRIP 1 – Buildings 75, Monash University, Wellington Rd., Clayton, Australia, 3800

## Abstract

In mammalian development, primordial germ cells (PGCs) represent the initial population of cells that are committed to the germ cell lineage. PGCs segregate early in development, triggered by signals from the extra-embryonic ectoderm. They are distinguished from surrounding cells by their unique gene expression patterns. Some of the more common genes used to identify them are *Blimp1, Oct3/4, Fragilis, Stella, c-Kit, Mvh, Dazl* and *Gcna1*. These genes are involved in regulating their migration and differentiation, and in maintaining the pluripotency of these cells.

Recent research has demonstrated the possibility of obtaining PGCs, and subsequently, mature germ cells from a starting population of embryonic stem cells (ESCs) in culture. This phenomenon has been investigated using a variety of methods, and ESC lines of both mouse and human origin. Embryonic stem cells can differentiate into germ cells of both the male and female phenotype and in one case has resulted in the birth of live pups from the fertilization of oocytes with ESC derived sperm. This finding leads to the prospect of using ESC derived germ cells as a treatment for sterility. This review outlines the evolvement of germ cells from ESCs *in vitro* in relation to *in vivo* events.

## Introduction

Recent advances in stem cell research have raised the possibility of differentiating germ cells in vitro from a starting population of embryonic stem cells (ESCs; Hubner et al. 2003; Toyooka et al. 2003; Geijsen et al. 2004; Lacham-Kaplan et al. 2006; Nayernia et al. 2006). These germ cells may theoretically be used as a treatment of infertility. Preliminary studies on mice have already resulted in offspring being obtained from the fertilization of oocytes with *in vitro* derived sperm (Nayernia et al. 2006). The offspring, however, displayed abnormalities, resulting in premature death. In order to replicate germ cell development *in vitro*, further understanding and in depth analysis into the tissue interactions and molecular mechanisms involved in germ cell differentiation *in vivo* is necessary.

As current research focuses on the mouse model, this review will focus on events leading to murine germ cell differentiation *in vivo* and *in vitro*, in relation to gene expression at specific embryo development stages and gender.

## Murine Germ Cell Development *In Vivo*

Separation of the germ line from the soma occurs early during mouse embryogenesis (Ginsburg et al. 1990). This early separation ensures that genetic or regulatory modifications that occur within the somatic cells during development have no effect on gamete formation, and in this way, are not passed on to the next generation. The exact manner in which germ line segregation occurs remains unclear; however, numerous genes have been implicated in regulating this segregation, in addition to regulating the migration and differentiation of these early germ cells.

### Stages of embryology in relation to germ cell development

#### Embryonic day 0.5–6.5—blastocyst formation and gastrulation

As a result of fertilization, two haploid cells merge to form a single diploid cell known as a zygote. This single cell proceeds through a number of mitotic divisions known as embryonic cleavage to form a morula which consists of approximately 8–16 cells. Further cell division leads to the formation of a blastocyst which is made up of two distinct layers. The outer epithelial cell layer, known as the trophectoderm, and a cluster of cells attached to one side of the inside surface of the trophectoderm, known as the inner cell mass (ICM). The remainder of the blastocyst is taken up by the fluid filled blastocoel cavity (Gardner et al. 1988).

By E5.5, the embryo is made up of the inner epiblast and the outer visceral endoderm (Tam et al. 1997). During the process of gastrulation, there is restructuring of the embryo morphology so that cells along the vegetal pole begin to flatten and are drawn into the interior, replacing the blastocoelic cavity. The three germ layers, the mesoderm, endoderm and ectoderm, are formed during this stage of development. The endoderm develops into the digestive tract; the ectoderm, into skin and the central nervous system and the mesoderm develops into the internal organs.

Gastrulation also involves the recruitment of epiblast cells to a transient embryonic structure called the primitive streak (Tam et al. 1993). One subset of these epiblast cells move towards the distal part of the extraembryonic region and form the allantois. Another subset of these epiblast cells move to the apical tip in the extraembryonic mesoderm and form the initial cluster of PGCs at E7.25 (Lawson et al. 1994; Okamura et al. 2003).

The manner in which PGCs are distinguished from the surrounding epiblast cells is unclear. Early experiments have suggested the existence of precursor PGCs within the epiblast cell population. These studies involved single cells being injected with a fluorescent dye at E6–6.5 and its descendants being traced after culture (Lawson et al. 1994). PGCs in the embryo showed fluorescence only when cells in the proximal one fifth of the epiblast were injected with the dye. These results suggest that a precursor PGC population may exist within the epiblast. In contrast, others suggest that environmental factors within the proximal region influence the fates of the epiblast cells (Tam et al. 1996). Tam et al. (1996) transplanted clumps of 5–20 cells of the distal epiblast at E6.5 into the proximal region of a recipient epiblast. Grafted cells that normally differentiate into ectoderm behaved like proximal epiblast cells with some differentiating into PGCs, overruling the predetermination of cells theory in early gastrulating embryos.

#### Embryonic day 7–8—primordial germ cells first appear

Primordial germ cells are destined to give rise to the entire population of mature germ cells of the organism. They start off as a cluster of about 45 PGCs that first appear in the mouse embryo at E7.25 at the base of the allantois, within the posterior extraembryonic mesoderm (Ginsburg et al. 1990; Lawson et al. 1994).

PGCs can be histologically recognized in early developing embryos by their large size, low nucleo-cytoplasmic ratio, clear nuclear borders, and granular nuclear chromatin. They are also identified by their position in the embryo, the presence of alkaline phosphatase activity and the presence of specific proteins that are unique to PGCs. The most common proteins are the transcription factor Oct3/4, the cell surface protein SSEA1, the nuclear protein Stella and the transmembrane protein Fragilis (Ginsburg et al. 1990; Sato et al. 2002; Saitou et al. 2002).

The combined action of bone morphogenetic protein 4 (BMP4) and BMP8b, both of which are expressed in the extraembryonic ectoderm (Yoshimizu et al. 2001), appear to be a critical element in PGC specification (Lawson et al. 1999; Ying et al. 2001). Inactivation of either gene will result in the lack of PGCs and allantoic tissue (Ying et al. 2001; Chuva de Sousa et al. 2004). BMPs are members of the transforming growth factor β (TGFβ) superfamily. The receptor involved in BMP8b signaling in PGC segregation is currently unknown, however, BMP4 is thought to act through the type I receptor ALK3, which upon ligand binding is phosphorylated by the type II receptor TALK (ten Dijke et al. 1994; ten Dijke et al. 1996; Hogan, 1996; Heldin et al. 1997). In PGCs, BMPs mediate signaling of Smad1, Smad4 and Smad5 proteins (Hayashi et al. 2002; Tremblay et al. 2001; Chang et al. 2001), which in turn act to regulate the transcription of target genes (Miyazono et al. 2000).

#### E8–13.5—Migration and proliferation of PGCs

PGCs begin migrating into the embryonic mesoderm at E8.5. They continue to migrate through the hindgut endoderm at E9.5, along the developing dorsal mesentry at E10.5, until finally they colonize the gonadal ridges at E12.5 (Wylie et al. 1985). Whilst migrating, they also proliferate and increase in number from about 100 at E8.5, to 1000 at E10.5 and to 4000 at E12.5. PGCs continue to proliferate after their arrival at the gonadal ridge where they reach about 25,000 PGCs at E13.5 (Godin et al. 1990; Tam and Snow, 1981). PGCs appear to autonomously regulate their growth timing; *in vivo* PGCs stop proliferating at E12–13, similarly *in vitro*, PGCs will also stop proliferating at the time corresponding to E12–13 (Ohkubo et al. 1996).

It is hypothesized that PGC migration is controlled by a repulsion mechanism involving the proteins Fragilis and Fragilis3 (described further in *fragili*s section). Germ cells expressing *fragilis3* are repelled from surrounding cells expressing *fragilis*. However, if both *fragilis* and *fragilis3* are expressed by the germ cells, then the repulsion effect is inactivated (Tanaka et al. 2005). Thus repulsion is only exerted when germ cells, which originally express both *fragilis* and *fragilis3*, stop expressing *fragilis* but continue to express *fragilis3*. Furthermore, RNAi silencing of *fragilis* results in prevention of the migration of PGCs out of the mesoderm. Once in the gonadal ridge, PGCs lose their motile behaviour (Wylie et al. 1985).

#### Embryonic day 13.5–17—Differentiation to germ stem cells and mitotic arrest

While PGCs become established in the gonad, they differentiate into germ stem cells (GSCs) that divide by mitosis to produce the supply of gametes that the organism requires for reproduction. Also known as A spermatogonia in the male, these single cells either renew themselves or produce Apr (paired) spermatogonia predestined to differentiate into mature sperm (De Rooij et al. 2001).

In the female, once mitotic proliferation stops, germ cells at E13.5 initiate prophase of meiosis. This phase is maintained until around 5 days after birth, where they progress to the late diplotene stage of the first meiotic division, and again go into arrest. Further maturation is then continued in adult females with each reproductive cycle. In males, germ cells arrest in the G_0_/G_1_ stage of the mitotic cell cycle (McLaren, 1984). Meiosis is not initiated until after birth.

In both males and females, meiosis is initiated by the expression of the *stimulated by retinoic acid 8 (stra8)* gene, who’s expression in turn is stimulated by retinoic acid (Koubova et al. 2006). Retinoic acid is provided by the mesonephroi of both sexes at E13.5. However, in the testis meiosis is retarded by the action of a retinoid-degrading enzyme known as CYP26B1. In the absence of CYP26B1, male germ cells enter meiosis prematurely as in a normal ovary (Bowles et al. 2006).

### Germ cell specific genes and their role in germ cell development

PGCs are traditionally distinguished in the embryo by their characteristic tissue non-specific alkaline phosphatase (TNAP) staining. TNAP staining, however, is not specific to PGCs since it is also expressed in the surrounding somatic cells (Hustin et al. 1987; MacGregor et al. 1995) and is thus not a reliable marker of PGCs. Finding a marker purely specific to PGCs has proven difficult, given that many of the genes expressed in PGCs are also expressed in their surrounding cells and they appear to be involved in maintaining or regulating pluripotency in PGCs. As such, these genes are also expressed in embryonic stem cell (ESC) and embryonic germ cell populations. Some of the most common germ cell genes used to identify PGCs are described in greater detail below.

#### Blimp1

Until recently the expression of *Fragilis* and *Stella* at E7.25 were thought to be the earliest markers of PGCs in the embryo. Recent studies have identified an earlier marker known as B-lymphocyte-induced maturation protein-1 (Blimp1) which is expressed at E6.25 in the first cell layer of the posterior proximal epiblast. Subsequently *Blimp1* is now thought to be the earliest gene to identify PGCs in the embryo (Ohinata et al. 2005; Vincent et al. 2005).

*Blimp1* encodes a transcriptional repressor, originally identified for its role in regulating plasma cell differentiation (Turner et al. 1994; Shaffer et al. 2002; Shapiro-Shelef et al. 2003). The protein structure implicates it has a role in gene expression regulation. At the N-terminus there is a positive-regulatory domain involved in chromatin remodeling, in the middle there is a proline rich region capable of recruiting transcriptional co-repressors (Ren et al. 1999; Yu et al. 2000), whilst at the C-terminus there are five consecutive Krupple-type zinc fingers for DNA binding. In PGCs, *Blimp1* functions by forming a complex with the arginine methyltransferase Prmt5. This complex is believed to be important in germ cell specific epigenetic programming (Ancelin et al. 2006).

*Blimp1* expression in the embryo is detected before gastrulation, at E6.25, in about four to eight cells located in a single layer in the most proximal epiblast cells (Table 1; Ohinata et al. 2005; Raz, 2005). Considerable increases in *Blimp1* expression follow, to around 16 cells at E6.5, 20–28 cells at E7.25, and approximately 40 *Blimp1* positive cells at E7.5 (Ohinata et al. 2005). Expression of *Blimp1* persists in PGCs as they migrate towards the genital ridges (Chang and Calame, 2002).

Results of Knock-out studies show a dose-dependant effect of *Blimp1* on PGC numbers. The study by Ohinata et al. (2005) observed a reduction in PGC number, ranging from 25 in wild-type; to 17.5 in heterozygotes; to five in *Blimp1* null, E7.5 embryos. Vincent et al. (2005) also observed a reduction in PGC numbers, from forty in wild-type, to nine in heterozygotes, and they did not detect any PGCs in *Blimp1* mutant embryos. Both of these studies found that PGCs from wild-type and *Blimp1* mutants can expand at similar rates, and thus both suggested *Blimp1* involvement in the initial specification of PGCs, rather than on their survival or proliferation (Ohinata et al. 2005; Vincent et al. 2005).

#### Oct3/4

Octamer-binding transcription factor 3/4 (Oct3/4) was originally identified as an embryonic stem cell (ESC) and germline specific marker (Okamoto et al. 1990; Rosner et al. 1990; Scholer et al. 1990). It is currently known to be a transcription factor belonging to class V of the POU (Pit-Oct-Unc) transcription factor family (Ryan et al. 1997). Members of this family have in common a bipartite DNA binding domain containing the POU homeodomain and the POU specific domain. The DNA sequence to which Oct3/4 binds in order to regulate gene expression is referred to as the octamer motif (Scholer et al. 1991). Upon binding of Oct3/4 to an octamer motif, transcription will either be initiated or repressed, depending on the flanking sequence (Pesce et al. 2001).

The expression of *Oct3/4* is regulated by a proximal promoter, a germ cell specific distal enhancer and a retinoic acid responsive element. In the epiblast, *Oct3/4* expression is driven by the proximal promoter. At gastrulation *Oct3/4* expression is down regulated and is maintained only in the germ cell lineage. *Oct3/4* expression is maintained in the germ cell lineage by the binding of germ cell specific, germ cell nuclear factor (GCNF) to the distal enhancer. Thus *Oct3/4* expression continues in germ cells under the regulation of GCNF (Fuhrmann et al. 2001). On binding of retinoic acid to the retinoic acid responsive element, *Oct3/4* expression is inhibited, allowing for lineage specific gene expression and differentiation of cells.

*Oct3/4* expression is not specific to germ cells; rather it is considered a marker of pluripotency. Expression is seen in oocytes, the inner cell mass, early pre-implantation embryos, primitive ectoderm, PGCs, embryonic stem cells, embryonic germ cells and embryonic carcinoma cells (Niwa, 2001). *Oct3/4* is also expressed at high levels in germ cell tumours (Looijenga et al. 2003). In the oocyte, *Oct3/4* functions as a maternal effect gene. Both the maternal RNA and the maternal protein are present in fertilized oocytes until the two cell stage, with zygotic expression taking over at the four to eight cell stages (Scholer et al. 1990; Palmieri et al. 1994; Yeom et al. 1996). *Oct3/4* expression decreases as the embryo develops, first in the outer cells of the morula as they form the trophectoderm and then in cells of the epiblast during gastrulation (E7.5), until it is eventually confined only to the PGCs of the embryo (Yeom et al. 1996).

PGCs, of both males and females, continue to express *Oct3/4* as they proliferate and migrate to the genital ridges (Table 1). In the male, expression in the germ cells persists throughout fetal development, and is maintained postnatally in proliferating gonocytes, pro-spermatogonia and in undifferentiated spermatogonia (Pesce et al. 1998; Tadokoro et al. 2002). However, *Oct3/4* expression is repressed in female germ cells at meiotic prophase I (E13–E14), and is then re-expressed after birth during the growth phase of the oocytes (Pesce et al. 1998; Tadokoro et al. 2002).

The results of *Oct3/4* knockout studies confirm its importance in early embryogenesis, particularly in ICM formation. Mutant blastocysts consist only of trophectoderm, and die between implantation and egg cylinder stages (Nichols et al. 1998). As *Oct3/4*-null embryos do not survive past such early developmental stages, they can not be used to examine the role of *Oct3/4* in PGCs. However, in studies where *Oct3/4* was ablated in PGCs during the migratory stage, PGCs became apoptotic (Kehler et al. 2004), suggesting an involvement of *Oct3/4* in PGC survival. Studies utilizing ESCs to investigate *Oct3/4* function found that forced over-expression of *Oct3/4* produced primitive mesoderm and endoderm (Niwa et al. 2000), while down regulation produced trophoblast like cells (Niwa et al. 2000; Velkey et al. 2003; Sen et al. 2004) and intermediate expression maintained the pluripotency of the ESCs in culture (Niwa et al. 2000).

#### Fragilis

*Fragilis* is a member of the interferon-induced transmembrane protein gene family (Saitou et al. 2002; Tanaka et al. 2002; Lange et al. 2003). Within this family are five related proteins composed of 104–144 amino acids, containing two transmembrane domains (Lange et al. 2003). Three of the members known as *fragilis, fragilis2* and *fragilis3,* appear to be associated with germ cells, while the remaining two members, *fragilis4* and *fragilis5*, are not detected within germ cells (Lange et al. 2003). The proteins reside on the cell surface membrane, with the amino and carboxy terminus ends being located outside of the cell.

*Fragilis* appears to be expressed in response to BMP4 signaling from the extra-embryonic ectoderm (Saitou et al. 2002). This is supported by the fact that *fragilis* positive cells are not detected in *BMP4*-null embryos, and are significantly reduced in *BMP4* heterozygous embryos (Lawson et al. 1999).

*Fragilis* expression occurs early in development, being detected throughout the epiblast in E6.0 embryos (Table 1). The expression of the marker becomes restricted at E6.5 to a cluster of cells at the posterior end of the embryo. The expression of *fragilis* in PGCs at this time is believed to be important for the migration of PGCs towards the genital ridges.

#### Stella

The *Stella* gene codes for a protein of 150 amino acids that is detected in both the nucleus and the cytoplasm of pre-implantation embryos in addition to the germ line (Saitou et al. 2002). The protein possesses a nuclear export signal, allowing it to shuttle between the nucleus and the cytoplasm. The N-terminus contains a modular domain which is similar to the SAP (SAF-A/B, Acinus and PIAS) motif; a DNA-binding domain that is involved in chromosomal organisation (Aravind et al. 2000). The C-terminus contains a splicing factor like motif, suggesting a role for Stella in RNA metabolism. In addition, the Stella protein is highly basic, allowing it to bind to both RNA and DNA (Aravind et al. 2000). In light of this the Stella protein is believed to be involved in chromosomal organisation and RNA processing and similar to other SAP-domain proteins, most likely has a role in chromosomal organisation by linking chromatin to RNA processes (Nayler et al. 1998; Aravind et al. 2000; Saitou et al. 2002). Stella doesn’t appear to have any function in the cytoplasm, but its storage there ensures it remains inactive. This is further manifested by experiments showing that forced over expression of Stella in somatic cells results in its containment within the cytoplasm (Saitou et al. 2002).

Stella is inherited as a maternal factor and is thus detected in oocytes and in zygotes (Payer et al. 2003). At the early morula stage, the maternally inherited Stella is degraded and replaced by the onset of zygotic expression (Payer et al. 2003). *Stella* expression persists until the blastocyst stage after which it is down regulated and doesn’t appear again until approximately E7.0 in 36–43 cells showing high *Fragilis* expression (Sato et al. 2002; Saitou et al. 2002). It continues to mark PGCs as they migrate through the hindgut at E8.5, and remains expressed in the germ line until about E13.5 in the female and E15.5 in the male (Table 1; Sato et al. 2002). Expression resumes within immature oocytes of newborn ovaries, and continues to be expressed in maturing oocytes; however no *Stella* expression is detected in adult testes.

Although *Stella* appears as a specific germ cell marker during early development, it doesn’t appear to be directly involved in PGC emergence (Payer et al. 2003; Bortvin et al. 2004). Stella knockout studies have revealed that first generation homozygous mutant mice contained germ cells comparable to those of wild-type or heterozygote mice. Spermatogenesis and ovarian follicle development appeared normal in these mutants and no morphological defects were detected (Bortvin et al. 2004). However, the second generations of mutant embryos die during the early cleavage stages (Bortvin et al. 2004), confirming the importance of *Stella* as a maternal factor involved in pre-implantation embryo development.

#### c-Kit and stem cell factor

C-Kit, a tyrosine kinase receptor, and its ligand stem cell factor (SCF; also known as Kit ligand or Steel factor), are key regulators of PGC growth and survival (De Miguel et al. 2002). Binding of SCF leads to dimerization of the c-Kit receptor, which causes the phosphorylation of different substrates depending on the activated pathway (Blume-Jensen et al. 1998). In PGCs, c-Kit is believed to be involved in activating the Protein kinase B or Akt (PKB/Akt) signaling pathway. However, unlike other cell types, activation of Akt does not appear to occur through PI3K (phosphatidylinositol 3-Kinase), but has been suggested to be activated by other c-kit downstream molecules such as the Sarcoma (Src) protein family (De Miguel et al. 2002). SCF exists as two forms, a membrane-bound growth factor, or as a soluble form resulting from proteolytic cleavage.

*C-Kit* is expressed in PGCs from their initial segregation at E7.5 (triggered by BMP4 signaling; Pellegrini et al. 2003) through to their arrival at the genital ridge at E13.5 (Table 1). In the mouse, *c-Kit* mRNA or protein are not detected in new born gonocytes, but expression reappears in differentiated spermatogonia and spermatocytes persisting until the round spermatid stage where it is no longer detected (Prabhu et al. 2006). Prabhu et al. (2006) observed that *c-Kit* mRNA could be detected in the absence of protein expression, indicating that transcription and translation of *c-Kit* are differentially regulated (Prabhu et al. 2006).

The importance of c-Kit and SCF in the germ cell pathway is highlighted by the finding that knock-out models are often completely sterile (Besmer et al. 1993; Lev et al. 1994), with similar effects observed in SCF mutants. Mice lacking membrane bound SCF are severely deficient in PGCs and are sterile (Dolci et al. 1991), even in the presence of soluble SCF (De Miguel et al. 2002).

During embryonic development, c-Kit is involved in the migration, proliferation, survival and differentiation of PGCs (Godin et al. 1991; Matsui, 1998; Kissel et al. 2000). SCF/Kit signaling is also important for the interaction between the SCF positive Sertoli cells and the c-Kit positive germ cells. Such interactions are required for the progression to the meiotic pachytene stage of spermatogenesis (Packer et al. 1995; Vincent et al. 1998).

The SCF/Kit pathway involvement in cell survival processes has also been demonstrated in ESCs (Palmqvist et al. 2005), where it is believed to function by suppressing apoptosis through the pro-survival protein Bcl-2 (Bashamboo et al. 2006). Over 80% of ESCs devoid of c-Kit signaling die by apoptosis when induced to differentiate (Bashamboo et al. 2006), which is significantly greater then the 30% seen in wild-type ESCs (Duval et al. 2000). The over-expression of *Bcl-2* appears to prevent cell death of differentiating ESCs (Duval et al. 2004). Although the SCF/Kit pathway appears to be required for the survival and differentiation of ESCs, it is not necessary for the differentiation of cells of the inner cell mass from which ESCs are derived (Geissler et al. 1981; Horie et al. 1991). Thus there appears to be a common role for c-Kit in regulating survival and differentiation of both ESCs and the germ cell lineage.

#### Mvh

The *mouse vasa homologue (Mvh)* gene encodes an ATP-dependant RNA helicase of the DEAD-box protein family (Hay et al. 1990). *Mvh* expression has not been detected in any tissue or embryonic cells (such as ESCs or EGCs) and appears to be exclusive to germ cells (Toyooka et al. 2000). In PGCs, *Mvh* expression is first expressed post migration, when PGCs have colonised the genital ridges, from E10.5–E12.5 (Table 1). It appears that a germ-soma interaction is necessary for *Mvh* expression. Its expression continues in germ cells until the spermatogenic cell and maturing oocyte stages in adult mice (Toyooka et al. 2000).

In the female, Mvh protein is located in the cytoplasm of developing oocytes. As the follicle matures, there is a decrease in Mvh until it is undetectable in mature oocytes (Toyooka et al. 2000). In the male, Mvh appears to be closely associated with chromatoid bodies of spermatocytes and spermatids. A granulo-fibrillar distribution of Mvh is seen in zygotene spermatocytes. By the pachytene-diplotene stage, Mvh aggregates into several granules and forms one large perinuclear granule in round spermatids at stage I–VII in the seminiferous tubules (Toyooka et al. 2000).

*Mvh*-null mice produce PGCs which exhibit decreased proliferative activity and in males result in defects in spermatogenesis. Hence *Mvh* knockout studies have resulted in sterility in males, while homozygous females are fertile (Tanaka et al. 2000).

Given that Mvh localisation coincides with the formation of the chromatoid body (a perinuclear granulo-fibrillar complex present in post meiotic cells, believed to function in post-transcriptional RNA metabolism), it is suggested that the two are connected (Toyooka et al. 2000).

#### Dazl

Dazl, a member of the DAZ family of proteins, is known to bind RNA and participate in translation of bound RNA by the recruitment of 80S ribosomes (Collier et al. 2005). Specific to the germ cell lineage, *Dazl* is first expressed in mice at E11.5 in post-migratory PGCs (Table 1; Seligman et al. 1998). In males, Dazl is situated in the cytoplasm of spermatogonia, pre-leptotene and zygotene spermatocytes, with the highest expression being found in pachytene spermatocytes (Ruggiu et al. 1997).

In both male and female mice, loss of *Dazl* results in the failure of germ cells to complete meiotic prophase (Saunders et al. 2003). Similar to the phenotype observed in *Mvh*-null male mice (Tanaka et al. 2000), *Dazl*-null male mice show a reduction in germ cell numbers and a failure of most cells to progress from A_aligned_ to A_1_ spermatogonia (Ruggiu et al. 1997; Schrans-Stassen et al. 2001), with few cells progressing to leptotene of meiotic prophase I (Saunders et al. 2003).

Ovaries of knock-out mice at E15 contain early pachytene oogonia (Ruggiu et al. 1997), however by E17.5 there is a large decrease in the number of germ cells with no more germ cells being present for four days after birth, resulting in infertile mice (Dekel, 1996; Ruggiu et al. 1997).

Given the results of these knock-out studies, the fact that Dazl binds *Mvh* mRNA in vitro and that *Dazl*-null mice show reduced levels of Mvh protein, it has been suggested that one of the functions of Dazl is assisting in the translation of *Mvh* in the male. It is doubtful, however, that Dazl is exclusive to *Mvh* translation as *Dazl*-null females are infertile, while *Mvh*-null females are fertile. Hence, Dazl must be involved in more than one process in the germ cell line.

#### Gcna1

Germ cell nuclear antigen 1 (Gcna1) is a nuclear antigen expressed in both male and female germ cells (Enders et al. 1994). *Gcna1* is first expressed in post-migratory PGCs (E10.5–E11.5), once they have entered the genital ridge (Table 1). Gcna1 continues to be present for the remaining embryonic period (Enders et al. 1994).

In females, Gcna1 remains in oocytes until 14 days post-partum, where it is no longer detected (Enders et al. 1994). In males, Gcna1 is detected in spermatogonia, leptotene and zygotene spermatocytes and spermatids. However more mature sperm located in the vas deferens and epididymis are not positive for Gcna1 expression (Enders et al. 1994).

Gcna1 appears to be very specific to germ cells. It has not been detected in any somatic cells tested to date, however, it has been detected in embryonal carcinoma cells. The function and role of Gcna1 in the germ cell lineage remains elusive.

## *In Vitro* Development Of Germ Cells

New advances in germ cell research have demonstrated germ cell differentiation in *in vitro* systems. Beginning with a population of embryonic stem cells, germ cells were shown to either spontaneously arise in culture (Hubner et al. 2003) or were induced to differentiate down a germ cell pathway, by the addition of growth factors (Nayernia et al. 2006) or by mimicking the early embryo environment in the form of embryoid bodies (Toyooka et al. 2003; Geijsen et al. 2004; Lacham-Kaplan et al. 2006). This *in vitro* system can potentially be used either therapeutically or as a research tool to enable a better understanding of germ cell differentiation. Germ cell differentiation *in vitro* may provide a model for lineage commitment and early populations of precursor cells that are often extremely difficult to access *in vivo.* Therapeutically, ESCs may in the future be used to cure or replace diseased or damaged tissue. ESCs may provide an unlimited source of specific cell types for transplantation, including reproductive cells.

### Embryonic stem cells

Embryonic stem cells (ESCs) originate from cells of the inner cell mass, collected from a developing blastocyst at E3.5 (Evans et al. 1981; Martin, 1981). Once removed from the embryo, these cells can be maintained in culture in the presence of embryonic fibroblast feeder cells and/or leukemia inhibitory factor (LIF; Smith and Hooper, 1987; Smith et al. 1988; Williams et al. 1988).

The maintenance of pluripotency and the self-renewal properties of ESCs are a result not only of their environment and culture conditions, but also a result of their own gene expression. Transcription factors identified to be important are the expression of *Oct3/4* (Pesce et al. 2001), *Nanog* (Chambers et al. 2003), *Sox2* (Avilion et al. 2003), and *foxd3* (Hanna et al. 2002).

Differentiation of ESCs occurs spontaneously upon the removal of feeder cells and LIF from the culture. Directing differentiation in a more controlled manner is most often achieved either by inducing formation of embryoid bodies (Boheler et al. 2002), adding certain growth factors to the cultures, nutrient restriction during ESC culture, or by the engineered expression of certain genes (Soria, 2001). Given their differentiation capabilities, ESCs are often used as the starting material by which to obtain cells of multiple lineages *in vitro*.

Germ stem cells can be obtained *in vitro* from ESCs cultured as monolayers or through the formation of embryoid bodies. Hubner et al. (2003), used an ESC line containing a PGC-specific *Oct3/4*-*GFP* transgene, where GFP (green fluorescent protein) expression is driven by a germ cell specific *Oct3/4* enhancer. These cells were cultured as a monolayer, without feeder cells or LIF, to allow for spontaneous differentiation. GFP expression was first evident after four days of culture. By day 7 of culture, 25% of the cells were positive for GFP as well as for Mvh. After 26 days of culture, cells resembling oocytes of 50–70 μm in diameter were present in the culture. These oocytes formed blastocyst like structures that expressed oocyte specific markers. In this study, oocyte differentiation occurred without additional growth factors, as cultures were supplemented only with serum and without any cellular organisation or niche formation (Hubner et al. 2003).

Nayernia et al. (2006) also uses monolayers to obtain germ stem cells. In this study haploid male gametes were differentiated from ESCs after three weeks of retinoic acid induction. These in vitro derived germ cells successfully fertilized wild-type oocytes and resulted in the full-term development of embryos and live post-natal offspring. However, the progeny displayed abnormalities, particularly in methylation patterns, and died prematurely.

### Embryoid bodies

Lineage specific differentiation can be controlled within embryoid bodies (EBs), derived from ESCs, to reflect that found in the embryo (Keller, 2005). EBs provide intimate intracellular contacts that mimic in vivo developmental niches. Analysis of EBs has demonstrated the differentiation of all three germ layers, ectoderm, mesoderm, and endoderm (Leahy et al. 1999). As differentiation continues, there is development of a range of cell types including cardiomyocytes (Klug et al. 1996), neural precursors (Brustle et al. 1997), and haematopoietic precursors (Potocnik et al. 1997).

Recent studies have demonstrated the ability of EBs to support the differentiation of germ cells *in vitro*. Geijsen et al. (2004) and Toyooka et al. (2003) obtained sperm like cells from mouse ESC derived EBs and Lacham-Kaplan et al. (2006) obtained oocyte like cells within ESC derived EBs.

In addition to the 3D supportive structure of the EBs, these studies also utilized the addition of growth factors to direct differentiation of germ cells. Toyooka et al. (2003) cultured EBs in the presence of BMP4 producing cells to mimic the *in vivo* system of germ cell differentiation. After one day in the presence of BMP4, Mvh positive cells were obtained from the EBs. When these cells were transplanted to the testis of sterile mice, they gave rise to sperm.

This study was soon followed by Geijsen et al. (2004) who cultured EBs in the presence of retinoic acid, which is known to rapidly differentiate ESCs while inducing the proliferation of PGCs. After around 20 days of culture, haploid male germ cells were obtained from the EBs. These cells gave rise to blastocysts when injected into oocytes.

EBs can also support the differentiation of oocytes. In the study by Lacham-Kaplan et al. (2006), EBs were cultured in the presence of testicular cell conditioned (TCC) medium that was prepared from the testis of 1 day old newborn male mice. After 2 weeks of culture in TCC medium, the EBs resembled ovarian structures that contained putative oocytes. Despite that a male ESC line was used to form EBs and that the EBs were cultured in testicular cell conditioned medium, female germ cells were obtained. An interesting difference between this study, where female germ cells were obtained, and the study by Toyooka et al. (2003) and Geijsen et al. (2004), where male germ cells were obtained, is that male germ cell differentiation resulted from cells that were sorted and thus separated away from surrounding cells, and either exposed to an *in vivo* male environment (Toyooka et al. 2003) or subject to male germ cell differentiation stimulus such as retinoic acid (Geijsen et al. 2004; Nayernia et al. 2006). Thus the isolation of cells differentiating down a germ cell pathway away from the other surrounding cells in the culture appears to be an important step if male germ cell differentiation is desired.

## Conclusions

Latest developments in ESC research have rendered possible the ability for the *in vitro* differentiation of ESCs into germ cells. Hence prospects of using ESC derived germ cells in regenerative medicine to overcome infertility related to germ cell deficiency are more feasible. A significant advancement in the field was the study by Nayernia et al. (2006) where they reported the birth of viable offspring from the fertilization of oocytes with *in vitro* derived sperm. Whilst this study provided a significant breakthrough in the field it also highlights the need for further research into this area given that the offspring displayed abnormalities resulting in premature death.

Studies on germ cell development hold exciting new solutions to assisted reproduction, yet more questions need to be addressed. Whilst nine genes were described in this review, many more are involved in germ cell characterisation and function and given the complexity of these cells the challenge for researchers will be to discover the role of many more. Further, given the nature of experiments in this field and the number of *in vitro* manipulations required, it will be essential to explore the accuracy of these findings in an *in vivo* setting, and more importantly with a functional read out, such as the birth of live, healthy pups with little or no abnormalities. In addition, a close investigation into the gene expression patterns displayed in *in vitro* differentiation, and whether this correlates with what is seen *in vivo* are necessary. It will also be important to use proteomics to assist in our understanding of the interaction between the soma and the germ line. One of the major limitations that researchers currently challenge is the paucity in our understanding of the *in vivo* maturation processes of germ cells. Thus the challenge lies in recapitulating germ cell differentiation and maturation *in vitro*. Collaborations between reproductive scientists, protein chemists and molecular biologists will be required if a complete understanding of germ cell development is to be obtained.

## Figures and Tables

**Table 1:** Expression patterns of PGC and GSC specific genes at different embryonic stages of germ cell development in vivo. + denotes expression at that developmental stage, − denotes no or very low expression.

	Pre-Migratory PGCs		Migratory PGCs		Post-Migratory PGCs			GSCs Female					GSCs Male				
Embryonic day	6.5	7.5	8.5	9.5	10.5	11.5	12.5	13.5	14.5	15.5	16.5	17.5	13.5	14.5	15.5	16.5	17.5
Blimp1	+	+	+	+	+	+	+	−	−	−	−	−	−	−	−	−	−
Oct3/4	+	+	+	+	+	+	+	+	+	−	−	−	+	+	+	+	+
Fragilis	+	+	+	+	+	+	+	+	+	+	+	+	+	+	+	+	+
Stella	−	+	+	+	+	+	+	+	−	−	−	−	+	+	+	−	−
C-Kit	−	+	+	+	+	+	+	+	−	−	−	−	+	+	−	−	−
Mvh	−	−	−	−	+	+	+	+	+	+	+	+	+	+	+	+	+
Dazl	−	−	−	−	−	+	+	+	+	+	+	+	+	+	+	+	+
Gcna1	−	−	−	−	+	+	+	+	+	+	+	+	+	+	+	+	+
